# Activating Mutations in *PTPN11* and *KRAS* in Canine Histiocytic Sarcomas

**DOI:** 10.3390/genes10070505

**Published:** 2019-07-04

**Authors:** Marilia Takada, Lauren A. Smyth, Tuddow Thaiwong, Marlee Richter, Sarah M. Corner, Peter Z. Schall, Matti Kiupel, Vilma Yuzbasiyan-Gurkan

**Affiliations:** 1 Comparative Medicine and Integrative Biology Program, College of Veterinary Medicine, Michigan State University, East Lansing, MI 48824, USA; 2 Veterinary Diagnostic Laboratory, College of Veterinary Medicine, Michigan State University, Lansing, MI 48910, USA; 3 Pathobiology & Diagnostic Investigation, College of Veterinary Medicine, Michigan State University, East Lansing, MI 48824, USA

**Keywords:** Bernese mountain dog, histiocytic sarcoma, *PTPN11*, *KRAS*, somatic mutation

## Abstract

While the genetic contributions to the predisposition of Bernese mountain dogs (BMDs) to histiocytic sarcoma (HS) remains unclear, some insights into key genetic drivers have been gained. Our group recently reported a mutation in the *PTPN11* gene (E76K). We have now identified a second missense mutation in PTPN11 (G503V), and a mutation in KRAS (Q61H) present in HS cell lines. These mutations are associated with malignancies in humans, and known to be gain-of-function mutations that result in activation of the mitogen-activated protein kinase (MAPK) pathway. The goal of the present study was to evaluate the prevalence of these mutations in a large sample of HS cases from BMDs and golden retrievers, and in lymphoma cases, from a cohort of BMDs. Mutations in *PTPN11* were present in HS in 41/96 (43%) BMDs, and in 3/13 (23%) golden retrievers. PTPN11 mutations E76K and G503V did not coexist in the same neoplasm. The *KRAS* mutation was much less frequent, with a prevalence of 3.1% (3/96). We did not identify either *PTPN11* nor *KRAS* mutations in any of the lymphoma samples. These results point out the potential relevance of *PTPN11* and *KRAS* mutations as activators of the oncogenic MAPK pathway for canine HS, particularly in BMDs.

## 1. Introduction

Histiocytic sarcoma (HS) in dogs is a highly aggressive neoplasm originating from malignant cells of dendritic cell lineage. It is a rare disease, accounting for less than 1% of all cancers in dogs, however it is frequently seen in certain breeds, especially in the Bernese mountain dog (BMD) which carries an incidence of more than 25%, and also in flat-coated retriever, golden retriever and Rottweiler [1,2,3,4,5,6,7,8]. There is no sex predilection for HS, and dogs in adult age (8–10 years) are most commonly affected [3,9]. HS develops most often in the skin, bone/joint, spleen, lymph node, lungs, and liver, where it rapidly disseminates to other organs in 70–91% of the cases [3,5,6,7,8,10]. Patients respond poorly to current treatment protocols, such as with lomustine and/or doxorubicin, which results in a median survival time of 3–6 months [3,8,11,12].

An analysis of genome-wide array comparative genomic hybridization carried out on samples from BMDs and flat-coated retrievers reported DNA copy number aberrations (CNAs) that are common to HS and these were shared in cases from both breeds. The most recurrent events were in tumor suppressor gene loci (*CDKN2A/B, RB1 and PTEN*) [13]. More recently, a genome-wide association study, conducted in a large cohort of HS in BMDs from North America and Europe, reported a common single haplotype in the same region of chromosome 11 containing genes *MTAP* and *CDKN2A*, which was present in 96% of affected dogs, and associated with HS [14]. However, the disease pathogenesis remains unclear.

The tyrosine-protein phosphatase non-receptor type 11 gene, or *PTPN11,* encodes SHP-2 (SRC homology-2 domain containing phosphatase), a non-receptor protein tyrosine phosphatase engaged in enhancement of signaling downstream of growth factors, cytokines and extracellular receptors. SHP-2 contains two SRC homology 2 domains (N- and C-SH2), a catalytic protein tyrosine phosphatase domain (PTP), and a C-terminal domain containing tyrosyl phosphorylation sites [15]. The N-SH2 domain interacts with the PTP domain, blocking its active site. Phosphotyrosyl peptide binding to the N-SH2 domain induces a conformational change that reverses the auto-inhibitory state [15].

In humans, PTPN11^E76K^ is the most common gain-of-function mutation seen in juvenile myelomonocytic leukemia (JMML), and was reported in acute myeloid leukemia (AML), while PTPN11^G503V^ was reported in AML and childhood leukemia [16,17,18,19]. Functional analyses demonstrated that these two variants led to a significant increase in the phosphatase activity level of SHP-2 and/or activation of downstream ERK and PI3K pathways, indicating their role as activating oncogenic mutations [16,19,20,21].

Interestingly, *PTPN11* mutations were also reported in four cases of human HS, a counterpart to the same disease in dogs. HS in humans is an extremely rare malignancy (<1% of all hematopoietic neoplasms) that is equally aggressive [22,23,24,25,26,27]. There is a lack of effective treatment options, and survival times rarely reach beyond one year [28,29]. Among the four individuals with mutated *PTPN11*, two carried the E76K, one carried the G503V, and one carried Y63S and Q506R variants [22,23,24,25].

**Figure 1:**
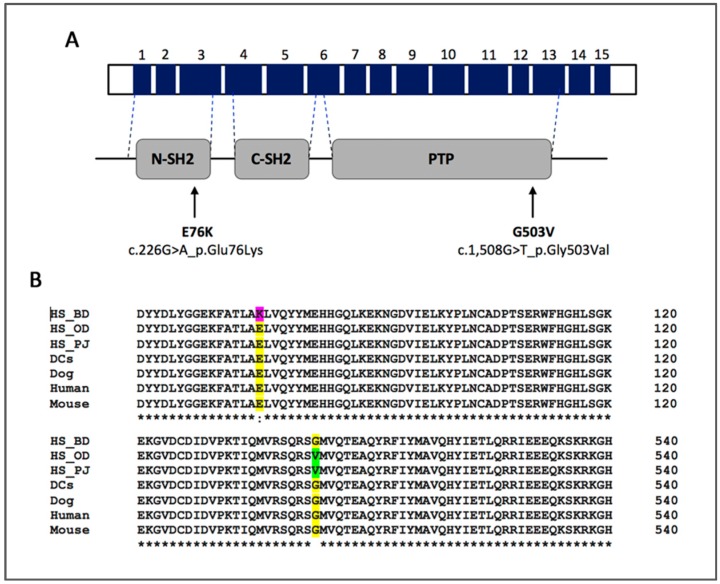
Sites of *PTPN11* mutations. (**A**) Schematic representation of *PTPN11* gene product with corresponding functional domains of SHP-2, indicating the locations of E76K and G503V mutations, with the predicted amino acid changes. Top bar is a schematic representation of the genomic structure with numbered exons (dark blue), and the lower bar depicts the functional domains. (**B**) Alignment of SHP-2 protein sequences from histiocytic sarcoma (HS) cell lines (BD, OD and PJ), normal canine dendritic cells (DCs), and reference sequences from relevant species (Dog, Human and Mouse). Missense mutations identified in canine HS, highlighted in pink (E76K) and green (G503V), are located within the highly conserved regions shown in yellow. Protein sequences were aligned using Clustal Omega software [31].

In canine HS, our group has recently reported the somatic mutation PTPN11^E76K^ on chr 26 in a cohort of HS from 30 BMDs and from 23 dogs of other breeds [30]. PTPN11^E76K^, a substitution mutation that is predicted to result in amino acid change (c.226G>A, p.Glu76Lys) located in exon 3 of the N-SH2 domain (Figure 1A,B), was present in 37% of HS from BMDs and 9% of HS from other breeds, indicating its potential as a driver mutation in canine HS, particularly in BMDs.

Our goal in the present study was to evaluate the prevalence of mutations within the MAPK pathway in a large cohort of BMDs with HS, as well as in a cohort of BMDs with lymphoma, the second most common malignancy in this breed. In addition to the previously reported mutations PTPN11^E76K^ and KRAS^Q61H^, we now report and include in the analysis a second missense mutation, PTPN11^G503V^ (c.1,508G>T, p.Gly503Val), located in exon 13 of the PTP domain of SHP-2 (Figure 1A,B) [32]. These mutations have recently been identified by our group in HS cell lines that have been established in our laboratory (two originating from BMDs and one from a Rottweiler), and DH82, a commercially available cell line through ATCC, originated from a hemophagocytic HS from a golden retriever, and originally described by Wellman et al. in 1998 [33] and further characterized by Heinrich et al. in 2015 [34]. In addition, we wanted to examine if there were any correlations with age, sex or clinical characteristics.

We screened a panel of HS specimens from BMDs and from golden retrievers, and confirmed that both *PTPN11* mutations are common in this disorder, particularly in BMDs, while the *KRAS* mutation was less prevalent. Additionally, none of these mutations are present in lymphoma samples of BMDs, suggesting that they may be especially relevant to HS, but not to lymphoma. Our findings contribute to a better understanding of oncogenic drivers of canine HS, indicating potential novel therapeutic targets for dogs, as well as for similar diseases in humans.

## 2. Materials and Methods

### 2.1. Subjects

Samples of HS from 32 dogs (19 BMDs and 13 golden retrievers) were obtained from archival formalin-fixed paraffin embedded (FFPE) tissues from the Veterinary Diagnostic Laboratory at Michigan State University (MSU). Samples from the 13 golden retrievers were previously reported [30].

Frozen tissues of 92 HS and 23 lymphoma cases from BMDs were selected from the BMD DNA and Tissue Repository initiative at MSU. Selection criteria included a confirmed diagnosis of the disease (HS or lymphoma) verified by a board-certified pathologist. These samples were left over tissues during treatment with surgical resection of the tumor, or post-mortem during necropsy, and were submitted by veterinary practitioners/dog owners to contribute to the BMD repository at our institution with signed consent. The use of left-over specimens was approved by the MSU Institutional Animal Care and Use Committee (IACUC), through an IACUC exemption process.

HS cell lines derived by our laboratory from BMDs (BD and OD), a Rottweiler (PJ) and a golden retriever (DH82, obtained from ATCC (CRL-10389)) were also included in the study. A comprehensive characterization of the BD, OD and PJ cell lines has been reported by our group [32,35]. DH82 is a commercially available cell line reportedly derived from a hemophagocytic HS in a golden retriever.

A blood sample, or normal tissue adjacent to the neoplasm in the paraffin block, was used to obtain constitutive DNA for each case. Blood samples were obtained from client-owned dogs and shipped with cold packs to MSU, where they were kept in aliquots at −80 °C. This procedure was approved by the MSU IACUC (AUF# 08/15-127-00). All dog owners provided written consent allowing the use of this material for research.

### 2.2. DNA Sample Acquisition

For genomic DNA extraction of the formalin-fixed paraffin embedded tissues, H&E slides were first marked around the areas containing neoplastic tissue. Samples of the marked areas were collected from the paraffin block with a scalpel blade. DNA extraction was performed using the RecoverAll Kit (AM1975, ThermoFisher, Waltham, MA, USA) following the manufacturer’s instructions. In the case of frozen tissue samples, HS cell lines and blood samples, genomic DNA was extracted from up to 25 mg of tissue, about 1 × 10^6^ cells or 100 μL of blood per sample using the DNeasy Blood & Tissue kit (69504, Qiagen, Germantown, MD, USA). Quantification of DNA was carried out using the Qubit dsDNA HS kit (Q33230, ThermoFisher) and a Qubit 2.0 fluorometer (ThermoFisher).

### 2.3. Methods for PTPN11 and KRAS Mutation Identification and Analysis

PTPN11^E76K^ and KRAS^Q61H^ were previously reported by our group [30]. More recently, we identified another mutation, *PTPN11*^G503V^, upon evaluating coding regions in RNA-seq data of our HS cell lines. For this purpose, messenger RNA was isolated, assayed for quality and sequenced using the Illumina HiSeq 4000 platform to generate a minimum of 150 million reads at 2X150 BP at the MSU Genomics Core. Data acquired from sequencing were checked for quality using FastQC and low-quality bases were trimmed using TrimGalore. *PTPN11* gene sequences of HS cell lines can be found under the Sequence Read Archive (SRA) study accession SRP139948. No other variants were identified within the coding region of *PTPN11* of the cell lines studied (BD, PJ and OD).

HS samples were genotyped using custom-made TaqMan SNP Genotyping Assays specific for the missense mutations c.226G>A (p.Glu76Lys) (Assay ID ANT2AD7, Lot P180405-000 B02), and c.1,508G>T (p.Gly503Val) (Assay ID ANDJ2M3, Lot P180403-001 E05) for the dog *PTPN11* gene; and for mutation c.183A>C (p.Gln61His) (Assay ID AN47YG4, Lot P180307-008 A08) for the dog *KRAS* gene (PN4332077, ThermoFisher). Using a 96-well plate, each reaction contained 0.5 μL of 20X TaqMan Custom SNP Assay, 5 μL of TaqMan Genotyping Master Mix (4371355, ThermoFisher), and 5 μL of genomic DNA 2 ng/μL. For each assay, two no-template negative controls containing DNAse free water, and three positive controls with known genotypes, as confirmed by Sanger sequencing, were included (homozygous/hemizygous mutant, heterozygous mutant and wild-type). Genotyping was conducted in real-time PCR mode using a StepOnePlus PCR System (4376600, ThermoFisher) following the cycling conditions instructed by the manufacturer. All assays were performed in duplicate experiments. Samples with ambiguous results were submitted for Sanger sequencing for confirmation using the primers 5′-TTT GTT TCC CCC TAA TGG AC-3′ (forward) and 5′-GAA CAC CTA TGG CAT GGA AGA-3′ [30] (reverse) for E76K, and 5′-CGA CAT TGA TGT TCC CAA AA-3′ (forward) and 5′-GGACAGCCATATAAATGAATCG-3′ (reverse) for G503V mutations in *PTPN11*, and 5′-TGG AGG AAT GTC TGT TCA GGT C-3′ (forward) and 5′-AAA ACA GGG GTA CAT TAC ATA CCT-3′ (reverse) for Q61H mutation in *KRAS*. A representative allelic discrimination plot is presented in the Supplementary Figure S1.

### 2.4. Statistical Methods

Categorical parameters including age (<5.5 years vs. 5.5–10 years vs. >10 years), sex (female vs. male), breed (BMD vs. golden retriever), and disease (HS vs. lymphoma) were statistically analyzed using a Fisher Exact Probability test or Chi-square test. *P* values were always given as a two-tailed analysis and were considered statistically significant for values below 0.05. All statistical analyses were performed using the VassarStats website [36].

## 3. Results

### 3.1. Cases Characteristics

One hundred and eleven cases of HS from BMDs were initially selected for the study, 15 cases were excluded due to the lack of available paired normal tissue, which resulted in the remaining 96 cases. These cases had histological confirmation of the diagnosis by a board-certified pathologist, and for a few, immunohistochemistry for CD18 was included in the diagnostic panel. Only for one case, the diagnosis was based on a cytology report (assessed by a veterinary pathologist), supportive clinical signs and diagnostics.

**Figure 2:**
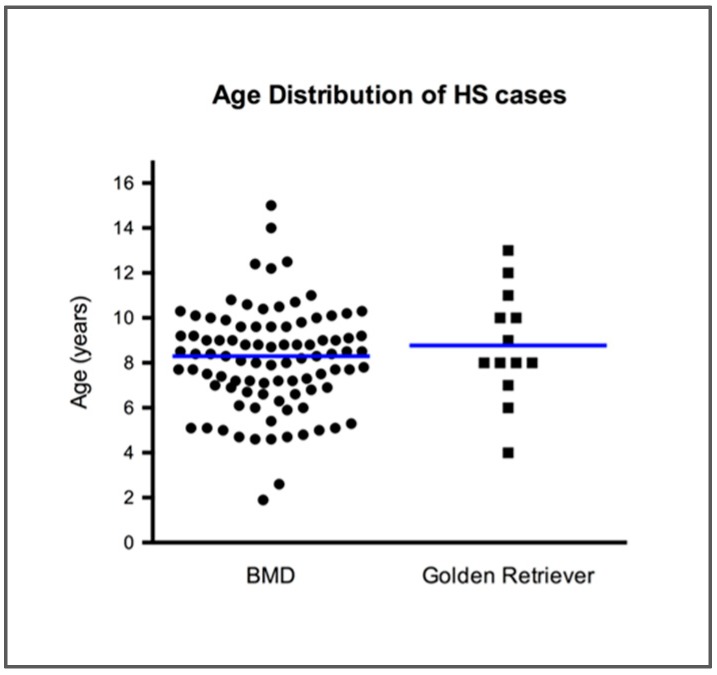
Age distribution in years of cases of histiocytic sarcoma (HS) from Bernese mountain dogs (BMDs) and golden retrievers. Blue horizontal lines represent the median age value.

Of the 92 cases with known age, the median age at diagnosis was 8.3 years (range: 1.9–12.5) (Figure 2). Forty-three female dogs, 50 male dogs and three dogs of unidentified sex were included in the study. Of 93 cases with reports of neoplasm location, at least two organs were affected with HS in 44% (41/93) of the cases in BMDs, with the most commonly affected organs being the lung (47/93), liver (32/93), spleen (32/93) and lymph node (19/93). Other locations affected at low frequency included mediastinum (7/93), stifle (6/93), peritoneum (4/93), urinary tract (4/93), intestine (4/93), skin/subcutis (4/93), cervical area (4/93), heart base (3/93), prostate (1/93) and pancreas (1/93). Our information was based on histopathology reports which were available for all cases, and clinical comments were included.

All HS samples from breeds other than BMDs also had histologically confirmed diagnoses. Among 13 golden retrievers with HS, the median age was 8 years (range: 4–13) (Figure 2), and six were female and seven were male dogs. The PJ cell line was derived from HS of an eight-year-old male Rottweiler [33]. The DH82 was reported to be derived from a hemophagocytic HS in a 10-year-old male golden retriever (ATCC (CRL-10389)). At least two anatomical locations were affected by HS in 23% (3/13) of the golden retrievers and the affected organs included the spleen (5/13), liver (4/13), skin/subcutis (4/13), lung (3/13), lymph node (3/13), peritoneum (3/13), pancreas (2/13) and stifle (1/13).

The BMD lymphoma cohort contained 23 lymphomas, from which 22 samples originated from lymph nodes, and one sample originated from the spleen. The diagnosis of malignant lymphoma was based on examination of the surgical biopsy by a boarded pathologist for fifteen cases (including the splenic sample) and eight cases were diagnosed based on cytology by a boarded clinical pathologist. The median age of this group at diagnosis was 8.4 years (range: <1–12.1), and among them, 13 were female and 10 were male dogs.

### 3.2. PTPN11 Mutation Status

*PTPN11* mutations were present in 41 of 96 (42.7%) HS from BMDs, 31 (32.3%) HS were positive for the E76K variant, and 10 (10.4%) for the G503V variant (Table 1). Interestingly, 12 of 41 (29.2%) HS with mutant *PTPN11* alleles were homozygous or hemizygous for the particular mutation, i.e., they did not have any wild-type allele. Additionally, there were no neoplasms that were compound heterozygous with regard to the mutant alleles.

The frequency of the PTPN11^E76K^ mutation in HS of BMDs previously published by our group (37% in 30 BMDs) did not differ from the prevalence of 32.3% that we found in this larger cohort of samples (*p =* 0.66) using a Chi Square Test with Pearson correction [30].

Across 13 HS cases of golden retrievers, *PTPN11* mutations were identified in three cases (23%). All three cases were heterozygous for the mutation, two (15.4%) were positive for the E76K variant and one (7.7%) for the G503V variant. There was no significant association between the presence of the mutation and breed of dogs (*p =* 0.1) based on a two-tailed Fisher Exact Probability test. The mutation status across all HS was stratified within groups of different ages and sex, and a two-tailed Fisher Exact Probability test was used to evaluate for any association between groups (Table 1). When the prevalence of the *PTPN11* mutation was compared across different ages, its presence was significantly associated with BMDs older than 10 years (*p =* 0.03). There was no statistically significant difference in the *PTPN11* mutation status between sexes (*p =* 1.0).

As previously mentioned, among the HS samples, we included four HS cell lines. We identified *PTPN11* mutations in all four cell lines: The BD cell line was positive for PTPN1*1*^E76K^, and OD, PJ and DH82 were all positive for the PTPN11^G503V^ mutation.

Interestingly, none of the 23 lymphomas from BMDs carried either one of the two *PTPN11* mutations studied. Therefore, when comparing HS and lymphoma from BMDs, the presence of a *PTPN11* mutation was significantly associated with HS (*p =* 0.0001) based on a two-tailed Fisher Exact Probability Test.

**Figure 3:**
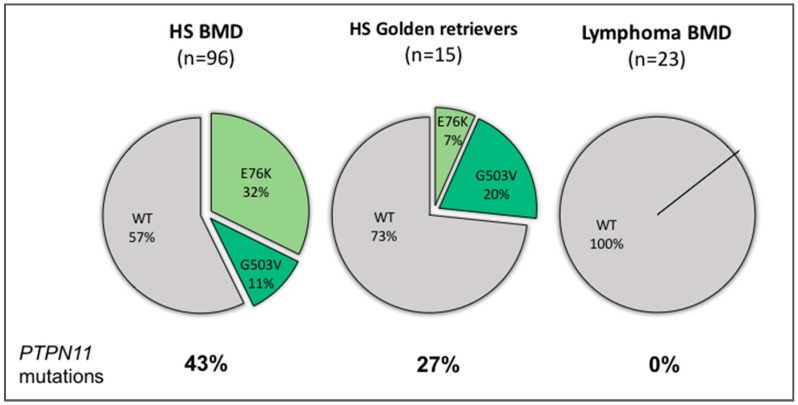
Frequency of tumor *PTPN11* mutation status based on breed, and disease type.

The results of the mutation status of dogs across the studied groups are presented in detail in Table 1, and schematically represented in Figure 3.

### 3.3. KRAS Mutation Status

The KRAS^Q61H^ mutation was present in 3 of 96 (3.1%) HS from BMDs, all three cases were heterozygous for the mutation. Two were male (ages 5.9 and 10 years) and one was a female BMD, (age 8.1 years) at diagnosis. This mutation was not present in any of the 23 lymphoma samples. In only one dog, KRAS^Q61H^ coexisted with a PTPN11 mutation (G503V). Across 13 HS cases of golden retrievers, none had the KRAS^Q61H^ mutation.

In this study, we only included tumor cases in which we had a normal paired tissue for comparison. Paired normal tissues from all cases were wild-type for *PTPN11* and *KRAS*.

## 4. Discussion

Our results demonstrated that somatic mutations in *PTPN11* are common in canine HS. *PTPN11* encodes SHP-2, the first reported mutant protein tyrosine phosphatase acting as an oncoprotein [16]. SHP-2 has a key role in signal enhancement in several signal transduction pathways associated with cell proliferation, differentiation and migration [37]. Qu and colleagues demonstrated that *PTPN11* function is also required for hematopoietic system development, and that the deficiency of SHP-2 in mice was lethal at mid-gestation with defects in mesodermal patterning [38].

We have recently identified and reported the frequency of the PTPN11^E76K^ mutation as 37% of HS in 30 BMDs [30]. In the present study, we have evaluated both the PTPN1*1*^E76K^ mutation and a second missense mutation, *PTPN11*^G503V^, in an expanded cohort of 96 cases of HS in BMDs. Our results show that the prevalence of the *PTPN11*^E76K^ mutation in this larger cohort of HS from BMDs is 32%, and not different from our previous findings (*p =* 0.66). While we found the PTPN11^G503V^ mutation to be present in only 10% of HS from BMDs, this increases the overall prevalence of *PTPN11* mutations to 43% in HS in BMDs, and emphasizes the oncogenic role of this particular gene in canine HS, specifically in BMDs. Constitutive DNA from cases from all cohorts were wild-type for both mutational spots in the *PTPN11* gene, demonstrating the somatic nature of this mutation.

In our cohort of BMDs with HS, most dogs were of adult age, and female and male dogs were equally represented, which is in agreement with historically reported data [3,4,7,39,40,41]. The prevalence of mutations was the same between sexes. On the other hand, mutation status was significantly associated with dogs older than 10 years compared to dogs of a younger age (*p =* 0.03). This may indicate that a specific genetic profile in younger dogs is sufficient to the development of HS, and do not require mutations in *PTPN11*. As mutations are acquired with age, this also may suggest that *PTPN11*-driven HS requires more prior mutations. Multiple organs were affected with HS in 44% (41/93) of the BMDs, from which 39% (16/41) carried *PTPN11* mutations.

Although the prevalence of *PTPN11* mutations was higher in HS of BMDs (43%) vs. golden retrievers (23%), there was no statistically significant association between the presence of the mutation and breed of dogs (*p =* 0.1). The lack of significance could be related to the small population of golden retrievers. We would require about twice the number of golden retrievers (*N* = 26) of equal *PTPN11* mutation frequency to achieve a *p*-value < 0.05 for the difference with BMD.

None of the lymphomas from BMDs had *PTPN11* mutations, indicating that within this breed of dogs, this mutation appears to be specifically associated with HS (*p =* 0.0001). However, we cannot exclude the possibility that similar mutations occur in other hematopoietic diseases, including other histiocytic diseases in BMDs, i.e., reactive histiocytosis, or other round cell neoplasm, i.e., mast cell tumors.

The gain of function mutations in *PTPN11* are associated with 35% of human JMML, a disorder with excessive proliferation of myelomonocytic cells, and have been reported in a low percentage of myelodysplastic syndrome (MDS) and AML cases, and a few cases of solid tumors [16,17,18]. Interestingly, identical mutations have also been reported in human HS [22,23,24,25]. Among the four human HS cases with mutated *PTPN11*, two carried the E76K variant, while one carried the G503V variant, and another carried Y63S and Q506R variants [22,23,24,25].

Similar to that seen in patients with JMML, MDS and AML, the *PTPN11* mutations in canine HS affected residues located at the N-SH2 (E76K) and PTP (G503V) interacting surfaces. Mutations in N-SH2 such as E76K, have been shown to cause a decrease in the binding affinity between the N-SH2 and PTP domains which affects the auto-inhibitory SHP-2 conformation, allowing access to the phosphatase catalytic site, resulting in a 5-fold increase of phosphatase activity when compared to wild-type SHP-2 [16,18,42]. In addition, a PTPN11^E76K^ mutation was shown to induce activation of the ERK and PI3K pathways through the increase and sustained interaction to Grb2, and the Gab2/p85 subunit, respectively [20]. In the case of PTPN11^G503V^, functional analysis reported by Niihori et al. demonstrated that the phosphatase activity was 2.7-times higher than wild-type SHP-2 [19].

The clinical implications of caring for canine patients with HS with mutations in *PTPN11* are not known. Clinical studies are warranted to understand whether neoplasms with or without *PTPN11* mutation behave differently clinically (i.e. with regard to rate of disease progression, rate of metastasis, and prognosis). In the current study, we do not have detailed information regarding the clinical course of the cases, and it will be important to monitor such variables as a response to different treatments, progress free survival and overall survival, and the mutational status of the tumor in future studies. It would also be important to understand whether neoplasms that are heterozygous, i.e., which contain one wild-type allele, are clinically distinct from those that appear homozygous for the mutant allele. In the current study, our assays did not allow us to differentiate hemizygosity from homozygosity for the mutant allele. It is possible that those neoplasms carrying two copies of the mutant allele, or zero copies of the wild-type allele would behave more aggressively. Interestingly, in our study, 29% (12/41) of the mutations were homo- or hemizygous and 71% (29/41) were heterozygous for the mutations. The percentage of homozygosity/hemizygosity might not be accurate in that it may be underestimated due to the possible presence of normal tissue in the tissue sections utilized, despite efforts to minimize this. Understanding the molecular mechanisms involved will certainly help in the management of affected patients, with the application of precision medicine to those canine patients.

Mutations in KRAS are present in 22% of all human cancers, and known to be activators of MAPK signaling [43]. Although KRAS^Q61H^ was rare in our study population (3/96), the same mutation was previously reported in a human HS by Liu et al. [23], another piece of evidence indicating a shared pathogenesis between similar diseases in humans and dogs. It is likely that there are other driver mutations that will be identified in canine HS. Interestingly, recent reports have identified additional mutations in a number of genes in the MAPK pathway in human HS [44] and targeted therapy aimed at inhibition of this pathway has yielded remarkably effective clinical results [45]. Certainly, these mutations arise in the context of other molecular changes in key genetic regions in the canine genome, as has been identified in previous studies [13,14].

Most importantly, our results open the venue to novel potential molecular therapeutic targets for HS. The development of SHP-2 inhibitors represents an active area of research. Compounds such as 11a-1, that binds to the SHP-2 activation pocket, and SHP099, that allosterically inhibits SHP-2, have demonstrated promising results both in experiments in vitro and in mouse xenograft models of cancer [21]. Downstream to SHP-2, MAPK and PI3K/AKT pathways are also potential targets for neoplasms with mutated *PTPN11* and *KRAS*, and for which several FDA-approved inhibitors are currently available. We have demonstrated that trametinib, an MEK inhibitor, significantly inhibited canine HS cells by promoting cell cycle arrest and cell apoptosis [32]. Additionally, trametinib prolonged survival of mice carrying xenograft HS orthotopically (intrasplenic) [33,46]. These findings suggest the role of an activated oncogenic MAPK pathway in canine HS. Clinical trials are needed to determine the association between *PTPN11*/*KRAS* mutation status and response to the inhibition of the MAPK pathway by trametinib. In addition, a more promiscuous kinase inhibitor, dasatinib, has also been shown by our group to be effective in prolonging the survival of mice in the same intrasplenic mouse xenograft model of canine HS [46].

In conclusion, we demonstrated that mutations in the *PTPN11* gene are commonly present in canine HS, especially in BMDs, indicating their important role as potential oncogenic drivers. Furthermore, these findings further highlight a targetable molecule and a key therapeutic pathway for HS in dogs and similar diseases in humans.

## Figures and Tables

**Table 1:** Frequency of tumor *PTPN11* mutation status based on breed, age, sex, and disease type.

Characteristics	*N*	*PTPN11* Mutant	*PTPN11* Wild-type	*P*	E76K	*P*	G503V	*P*
**HS Bernese mountain dogs**	96	41 (43%)	55 (57%)		31 (32%)		10 (10%)	
**Age**								
<5.5 years	14	6 (43%)	8 (57%)	NS	3 (21%)	NS	3 (21%)	NS
5.5–10 years	60	19 (32%)	41 (68%)		16 (27%)		3 (5%)	
>10 years	18	11 (61%)	7 (39%)	**0.03**	8 (44%)		3 (17%)	
Unknown age								
**Sex**								
Female	43	17 (39%)	26 (60%)	NS	13 (30%)	NS	4 (9%)	NS
Male	50	21 (42%)	29 (58%)		15 (30%)		6 (12%)	

**HS golden retrievers**	13	3 (23%)	10 (77%)	NS	2 (15%)	NS	1 (8%)	NS
**Lymphoma BMDs**	23	0	23 (100%)	**0.0001**	-		-	

NS: Non significant value

## Supplementary Materials

The following are available online at https://www.mdpi.com/2073-4425/10/7/505/s1, Figure S1: Representative allelic discrimination plot from a *PTPN11*^G503V^ genotyping assay.
