# Supplementary material for: Activating Mutations in PTPN11 and KRAS in Canine Histiocytic Sarcomas
Source: Genes (Basel). 2019 Jul 4;10(7):505. doi: 10.3390/genes10070505 (PMC6678586; doi:10.3390/genes10070505)

Supplementary Figure S1: Representative allelic discrimination plot from a PTPN11^{G503V} genotyping assay. Genotype is determined by sequence-specific probes, one for each allele (wild type and mutant). Each circle represents a sample, and black squares represent negative controls. Sample indicated as an X, represents an undetermined genotype, and further characterized by Sanger sequencing for determination of genotype.

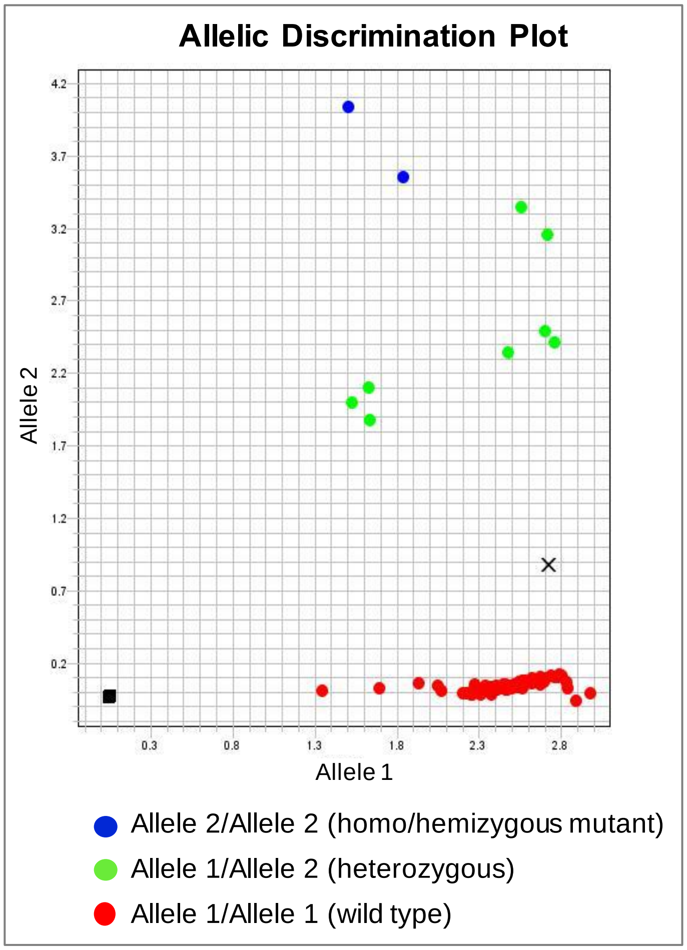

Supplement: Supplementary file 1 [file genes-10-00505-s001.pdf]
